# Prediction of Acute Kidney Injury in Oncology: From Clinical Risk Scores to AI-Enabled Precision Onco-Nephrology

**DOI:** 10.3390/cancers18182937

**Published:** 2026-09-10

**Authors:** Brena F. Sena, Alexandre Homo, Camille Fahy, Anthony Van de Putte, Bertrand Roudier, Adrien Ugon, Corinne Isnard Bagnis

**Affiliations:** 1 Department of Nephrology, AP-HP Sorbonne Université, 75013 Paris, France; 2 ESIEE Paris, Université Gustave Eiffel, 93162 Noisy-le-Grand CEDEX, France; camille.fahy@edu.esiee.fr (C.F.); adrien.ugon@esiee.fr (A.U.); 3 Laboratoire de Biomécanique Appliquée (LBA), UMR T24, Aix-Marseille Université, Université Gustave Eiffel, 51 Boulevard Pierre Dramard, 13015 Marseille, France; 4 European Renal Association Onconephrology Working Group

**Keywords:** acute kidney injury, artificial intelligence, machine learning, oncology, nephrotoxicity, onco-nephrology

## Abstract

**Background/Objectives:** Acute kidney injury (AKI) is a common complication across the cancer care continuum and can compromise kidney function, delay or interrupt anticancer therapy, and worsen renal and oncologic outcomes. This state-of-the-art narrative review examines conventional clinical risk scores and artificial intelligence (AI)- and machine learning (ML)-based approaches to AKI prediction in oncology and identifies priorities for clinical translation. **Methods:** We conducted iterative searches of PubMed and Google Scholar through July 2026 and screened reference lists of relevant primary studies and reviews. We prioritized oncology-specific model development and validation studies and selectively included conventional scores and observational evidence where dedicated prediction models were unavailable. **Results:** Prediction evidence is most developed in hospitalized cancer populations, cisplatin exposure, contrast-enhanced computed tomography, immune checkpoint inhibitor therapy, and selected oncologic surgical procedures. Hematopoietic stem cell transplantation includes an early conventional risk score, whereas evidence for CAR T-cell therapy and targeted therapies remains predominantly observational. Most studies are retrospective, and independent external validation, calibration assessment, fairness evaluation, and prospective workflow implementation remain uncommon. Reported performance cannot be compared directly across studies because outcome definitions, prediction windows, populations, and validation strategies differ. **Conclusions:** AI- and ML-based AKI prediction may support precision onco-nephrology, but no oncology-specific model has yet demonstrated improved outcomes in a prospective interventional study. Clinical progress will require standardized outcomes, treatment-aware longitudinal data, rigorous external validation, and prediction tools linked to evidence-based response pathways. These advances may ultimately enable precision onco-nephrology by supporting proactive kidney protection while preserving optimal cancer treatment.

## Simple Summary

Acute kidney injury is a frequent complication of cancer and its treatment. It may interrupt anticancer therapy, worsen long-term kidney function, and contribute to poorer outcomes. Artificial intelligence and machine learning methods could identify patients at increased risk before kidney injury becomes clinically apparent, allowing earlier monitoring and preventive care. This review examines prediction approaches across hospitalized cancer populations, chemotherapy, contrast-enhanced imaging, immune checkpoint inhibitors, stem cell transplantation, cellular therapy, targeted therapy, and oncologic surgery. Although several models show promising performance, most have been developed retrospectively, few have been externally validated, and none has demonstrated improved patient outcomes through prospective clinical implementation. Progress will depend on standardized definitions, longitudinal oncology data, transparent and equitable models, external validation, and integration with clearly defined clinical response pathways.

## 1. Introduction

The relationship between cancer and kidney disease has become increasingly important as advances in oncology continue to improve survival and expand treatment options. Modern cancer care relies on therapies that have transformed outcomes across many malignancies but frequently expose patients to substantial nephrotoxic risk. Platinum-based chemotherapy, targeted therapies, immune checkpoint inhibitors (ICIs), cellular therapies, contrast-enhanced imaging, and increasingly complex multimodal treatment strategies expose patients to diverse and often overlapping nephrotoxic risks throughout the cancer care continuum [1,2,3]. Preserving kidney function is therefore central not only for reducing morbidity but also for maintaining access to effective anticancer treatment [4,5].

Acute kidney injury (AKI) is particularly consequential because even modest deterioration in kidney function may lead to dose reductions, treatment delay, regimen modification, or discontinuation of anticancer therapy [4,5,6]. Treatment delays are associated with increased cancer mortality [7], while AKI may also lead to recurrent injury, chronic kidney disease (CKD), prolonged hospitalization, and poorer long-term outcomes [8,9,10].

The burden of AKI varies substantially by malignancy, treatment, and clinical setting. In a Danish cohort of 37,267 patients with cancer, the one-year cumulative incidence of AKI was 17.5%, with particularly high risk in multiple myeloma, liver cancer, and kidney cancer [11]. Approximately one in ten patients receiving systemic anticancer therapy developed AKI in a population-based Canadian study [12]. Among 482,016 older US adults with newly diagnosed cancer, the five-year cumulative incidence of AKI was 19.8%, with marked variation across cancer types [13]. Contemporary Danish data further demonstrate treatment-specific heterogeneity, ranging from less than 1% after breast cancer surgery to more than 50% after radical nephrectomy or allogeneic hematopoietic stem cell transplantation (HSCT), with AKI consistently associated with increased mortality [14].

Risk is even greater among hospitalized and critically ill patients, where sepsis, tumor lysis syndrome, hemodynamic instability, intensive chemotherapy, and nephrotoxic supportive care frequently coexist [9,15]. Many patients also enter treatment with reduced renal reserve. The IRMA study established that impaired kidney function is common at treatment initiation [16], and more recent oncology cohorts confirm a substantial prevalence of CKD across malignancies [17].

Cancer-associated AKI is often multifactorial, and may arise from the malignancy itself, urinary tract obstruction, volume depletion, infection, contrast exposure, direct treatment toxicity, or immune-mediated injury [1,2,3]. These mechanisms can occur simultaneously or sequentially as patients move between ambulatory therapy, imaging, surgery, and hospitalization. This complexity has contributed to the growth of onco-nephrology as a subspecialty focused on preserving kidney health throughout the cancer journey [18].

Current practice nevertheless remains largely reactive, typically occurring after a rise in serum creatinine or decline in urine output. For research and standardized classification, AKI is most commonly defined and staged according to Kidney Disease: Improving Global Outcomes (KDIGO) criteria [19]. Although KDIGO provides the accepted framework for AKI definition and severity classification, explicit staging is not consistently applied in routine clinical practice, and clinically significant AKI may remain underrecognized. By the time functional changes become apparent, meaningful kidney injury may already be established [20]. Advances in AI, ML, and electronic health record (EHR) analytics have created opportunities to identify elevated risk hours to days earlier in general hospital populations [21,22]. The March 2026 KDIGO AKI/AKD public-review draft emphasizes the potential role of externally validated clinical risk models when linked to kidney-protective care pathways [23].

The key question is therefore not simply whether AKI can be predicted, but whether prediction systems can capture the dynamic nature of kidney risk in cancer and translate earlier recognition into actions that preserve kidney function and treatment continuity. This review synthesizes conventional and AI/ML-based AKI prediction evidence across major oncology settings, places these studies within the broader AKI prediction literature, and identifies priorities for validation, implementation, and precision onco-nephrology.

## 2. Literature Search and Review Approach

This state-of-the-art narrative review was informed by iterative anchor searches of PubMed and Google Scholar conducted through 23 July 2026, supplemented by backward and forward citation searching and targeted searches across major oncology settings. Search concepts combined “acute kidney injury” or “AKI” with “cancer” or “oncology” and terms related to prediction, clinical risk scores, artificial intelligence, and machine learning. Additional targeted searches addressed cisplatin, contrast-enhanced imaging, immune checkpoint inhibitor, hematopoietic stem cell transplantation, CAR T-cell therapy, targeted anticancer therapy, and oncologic surgery. Searches were restricted to English-language publications, with no restriction on publication date. We prioritized peer-reviewed studies that developed or evaluated conventional or AI/ML-based AKI prediction models in cancer populations. Where dedicated models were unavailable, we considered cancer-specific observational studies reporting clearly defined AKI outcomes and clinically relevant risk factors. Studies were prioritized by author consensus according to clinical relevance, methodological contribution, population and treatment specificity, and contribution beyond evidence already represented. Complete search strategies are provided in Supplementary Tables S1 and S2. Consistent with the narrative-review design, no formal protocol, exhaustive duplicate screening, risk-of-bias assessment, or meta-analysis was undertaken. Evidence maturity was categorized descriptively as “emerging” when evidence consisted primarily of observational studies or initial model development without independent validation; “promising” when a dedicated prediction model had undergone internal, temporal, or limited external validation; and “mature” when evidence included independent external validation, prospective evaluation, and progress toward clinical implementation. These categories are descriptive author assessments and do not constitute a formal evidence-grading system (Supplementary Methods S1).

## 3. Why AKI Prediction Matters in Oncology

The rationale for oncology-specific AKI prediction is the possibility that some episodes may be prevented or mitigated when increased risk is recognized before clinically overt injury. Earlier identification may support individualized kidney health assessment, nephrotoxin stewardship, optimization of hydration and drug dosing, intensified monitoring, and timely nephrology involvement. These measures are most useful when initiated before injury is established, but prediction alone is unlikely to improve outcomes unless it is linked to a clearly defined clinical response pathway [24].

The clinical importance of prediction also varies according to severity and trajectory of AKI. Predicting a first episode of creatinine-defined AKI addresses a different clinical question from predicting recurrent AKI, severe AKI, kidney replacement therapy, persistent kidney dysfunction, or AKI that disrupts cancer treatment. Recurrent AKI may reflect cumulative treatment exposure, incomplete renal recovery, persistent vulnerability, or repeated clinical insults and may therefore require different predictors and intervention strategies from a first AKI event. Severe or recurrent AKI may be particularly important targets for risk stratification and may justify different thresholds for preventive intervention. AKI is associated with mortality, CKD, prolonged hospitalization, and greater healthcare utilization [24,25,26]; in oncology, it can also determine eligibility for nephrotoxic or renally cleared therapies. Cisplatin is a clear example, where even modest kidney dysfunction may trigger dose reduction, delay, or discontinuation and reduce cumulative treatment intensity [6,27]. Similar challenges occur with ICIs, targeted agents, cellular therapies, and multimodal regimens, although the relevant injury mechanisms and preventive options differ by treatment.

Risk stratification could therefore help direct limited resources toward patients most likely to benefit while avoiding unnecessary surveillance among lower-risk individuals. The most useful systems will likely update risk over time as patients accumulate treatment exposures, undergo imaging or surgery, develop infection, and receive supportive medications [22,28,29]. The aim is not to replace clinical judgement, but to provide timely and actionable information within precision cancer care [30].

### 3.1. Unique Features of AKI in Patients with Cancer

AKI in cancer is not simply a more frequent form of hospital-associated AKI. It is a heterogeneous syndrome shaped by cancer biology, treatment toxicity, immune dysregulation, recurrent infection, supportive care, structural complications, and pre-existing renal vulnerability. Treatment-related intrinsic kidney injury may occur alongside hemodynamic or infectious insults, while malignancy itself or cancer-directed procedures may cause postrenal AKI through urinary tract obstruction. These phenotypes have different risk factors, trajectories, and opportunities for prevention or intervention and should not necessarily be pooled within a single prediction framework.

Multiple insults often occur concurrently or sequentially. A patient receiving cisplatin may also have vomiting or diarrhea, receive nephrotoxic antibiotics, undergo contrast-enhanced imaging, or develop obstruction related to tumor burden or urinary tract involvement. Such combinations may produce overlapping prerenal, intrinsic, and postrenal mechanisms. Volume depletion is particularly common yet difficult to assess, and conventional indices such as fractional excretion of sodium have limited diagnostic performance in the presence of CKD or diuretic use [31,32].

The malignancy itself may directly impair kidney function through urinary tract obstruction, hypercalcemia, paraprotein-associated disease, tumor lysis syndrome, thrombotic microangiopathy, or paraneoplastic glomerular disorders [2,3,33]. Hematologic malignancies add further complexity through intensive chemotherapy, severe infection, HSCT, and disease-specific processes such as cast nephropathy [34,35].

Baseline kidney function is also frequently uncertain. Older age, hypertension, diabetes, previous AKI, and CKD reduce renal reserve, while cancer-related sarcopenia and cachexia can make serum creatinine misleadingly low. Recent guidance supports CKD-EPI-based estimates, creatinine-cystatin C equations when creatinine is unreliable and cystatin C when available and measured glomerular filtration rate in selected high-risk or borderline cases [4,36]. In practice, cystatin C and measured GFR remain inconsistently available, so some patients begin therapy with incompletely characterized kidney vulnerability.

The defining challenge for prediction is the dynamic relationship between kidney risk and cancer treatment. Successive chemotherapy cycles, immunotherapy, targeted therapies, recurrent imaging, surgery, supportive medications, infections, and hospitalizations all contribute to a continuously evolving risk profile. Combination and sequential regimens may generate additive, cumulative, or interacting effects that cannot be assigned to a single exposure. Population-based evidence showing wide variation across malignancies, treatments, and clinical settings supports viewing cancer-associated AKI as a spectrum of overlapping etiologic and treatment-related phenotypes rather than a single disease process [14].

**Figure 1:**
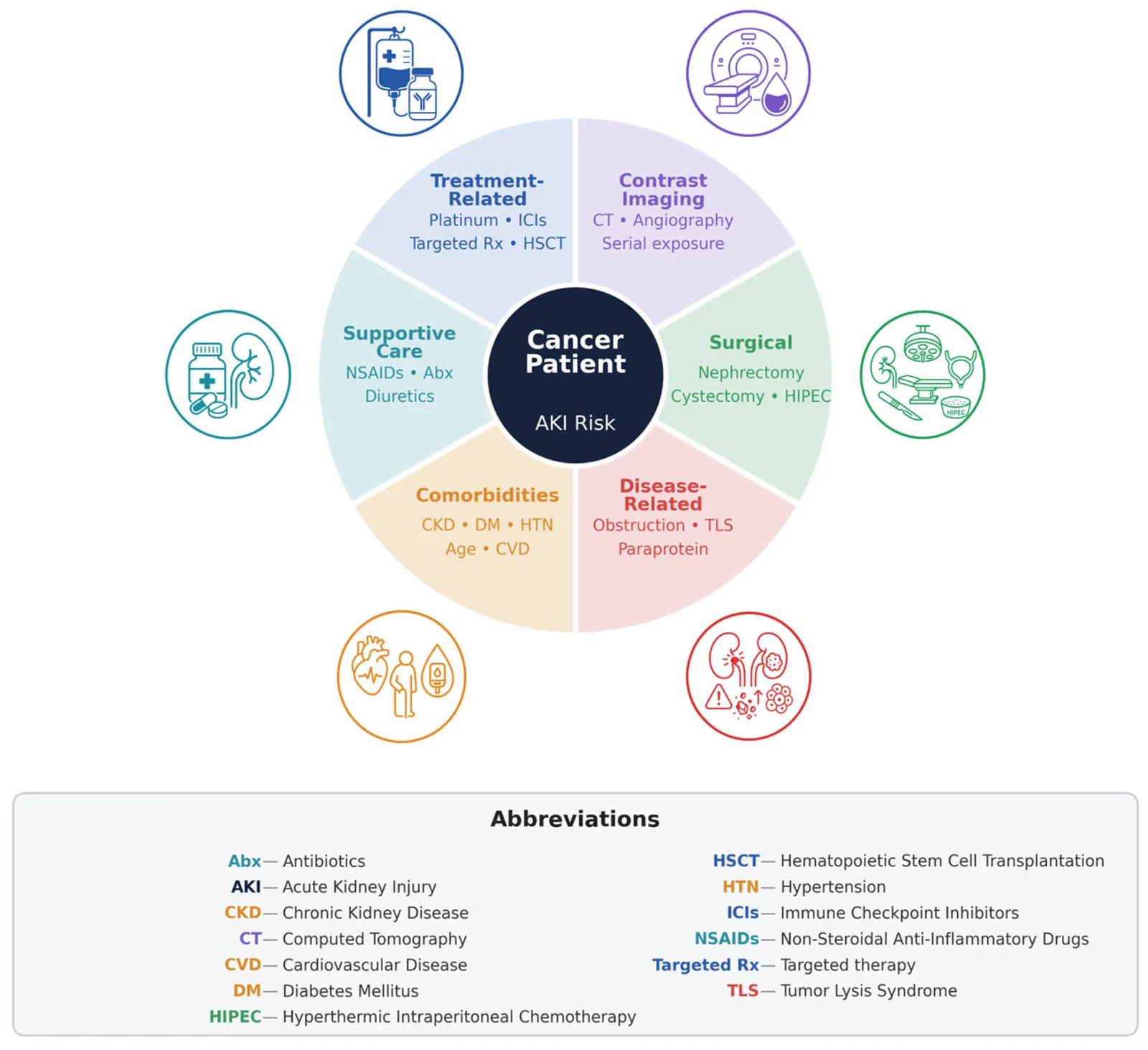
Drivers of AKI risk in oncology: Cancer-associated AKI reflects interacting patient, disease, treatment, supportive-care, imaging, and surgical factors that may accumulate across the cancer journey.

Most existing models nevertheless estimate risk at one baseline time point, before one treatment course, procedure, or imaging encounter. Such static estimates cannot capture cumulative dose, regimen sequencing, changing kidney function, or emerging complications. Effective prediction will require longitudinal treatment histories, laboratory trajectories, concurrent nephrotoxins, and evolving clinical status. Figure 1 summarizes the principal patient-, disease-, and treatment-related drivers of AKI risk and highlights why dynamic risk trajectories are central to precision onco-nephrology.

### 3.2. Evolution of AKI Prediction and Lessons for Oncology

Clinical AKI prediction has evolved from regression-based scores using a small number of predefined variables to ML approaches capable of analyzing high-dimensional and longitudinal EHR data. Early models offered transparency and ease of implementation but were derived in single-center cohorts and lacked external validation. A systematic review by Hodgson et al. identified substantial heterogeneity in predictor selection, baseline creatinine definitions, AKI outcomes, and validation strategies [37].

The expansion of structured EHR data enabled random forests, gradient-boosted trees, support vector machines, and neural networks to model nonlinear relationships and interactions across laboratory, medication, physiologic, and demographic variables. Wang et al. showed that ensemble learning could improve discrimination in imbalanced EHR datasets [38]. However, systematic reviews have consistently found that algorithmic sophistication does not compensate for weak design, heterogeneous outcomes, or inadequate validation [39,40].

Longitudinal modeling was a major conceptual advance. Tomašev et al. trained recurrent neural networks on more than 700,000 adults and predicted AKI up to 48 h before clinical recognition, demonstrating that temporal trajectories contain information unavailable from isolated measurements [22]. Churpek et al. subsequently showed that a simpler gradient-boosted model could achieve strong performance and external validity across nearly 500,000 admissions from three health systems [21]. These studies illustrate that scalable implementation may depend as much on parsimonious design and transportability as on model complexity.

More recent reviews have similarly concluded that complex ML methods do not consistently outperform carefully developed regression or ensemble models when trained on comparable data [39,41]. The principal determinants of clinical usefulness are high-quality data, appropriate prediction horizons, standardized outcome labels, calibration, external validation, interpretability, and integration into workflows. These lessons are directly relevant to oncology, where general inpatient variables such as vasopressor use may be informative during critical illness but not capture cumulative chemotherapy, cancer stage, treatment sequencing, or ambulatory nephrotoxic exposures.

**Figure 2:**
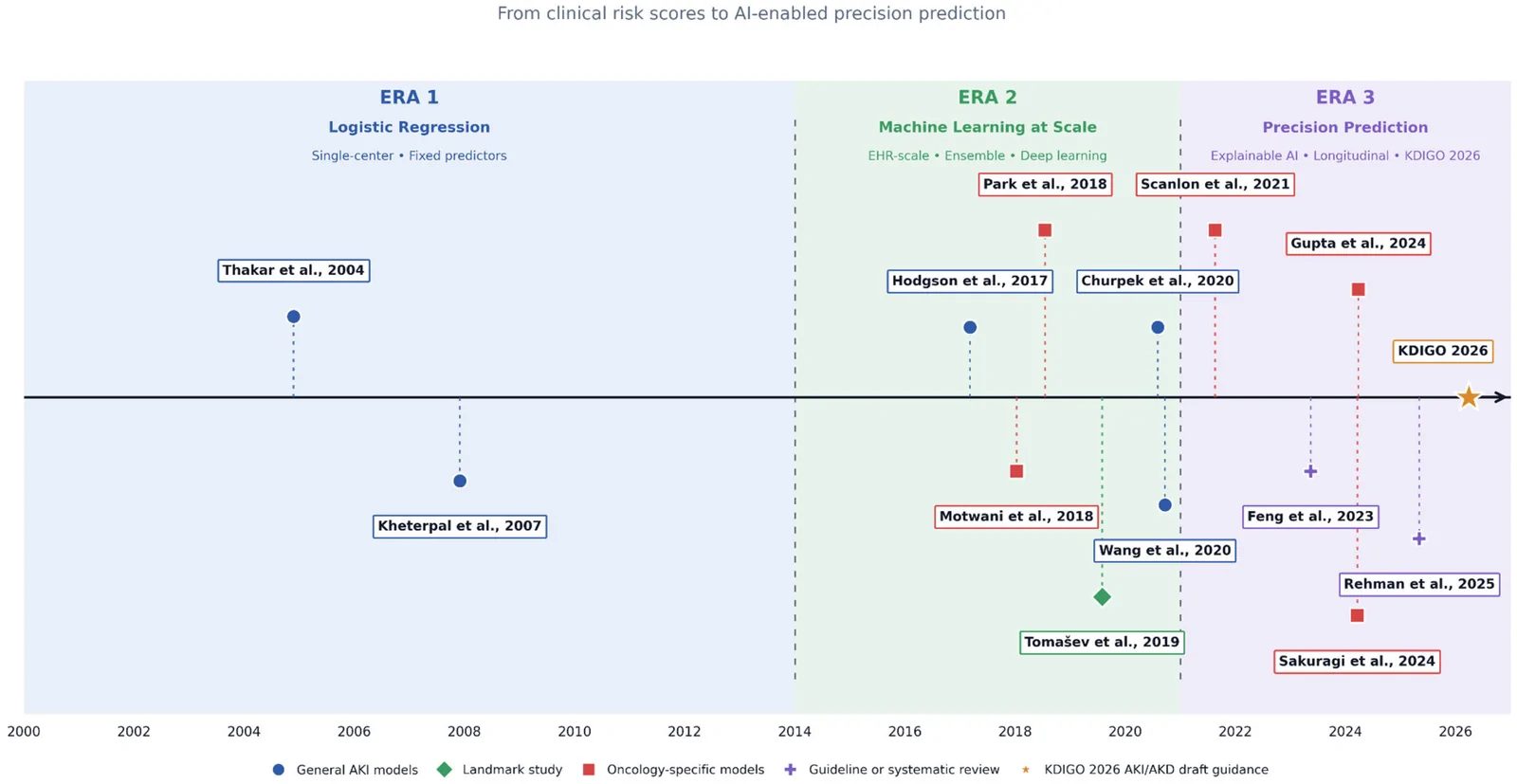
Evolution of AKI prediction: From regression-based clinical scores to longitudinal, externally validated, and explainable approaches. The timeline shows representative milestones rather than an exhaustive list [21,22,23,29,37,38,39,41,42,43,44,45,46,47]. KDIGO 2026 refers to the March 2026 AKI/AKD public-review draft, not a finalized guideline.

General AKI models therefore provide a methodological roadmap rather than a ready-made solution for cancer care. Oncology-specific systems must incorporate treatment context and changing risk while preserving calibration and clinical actionability. Figure 2 summarizes the transition from static clinical scores to longitudinal and explainable prediction, alongside representative milestones in oncology-specific research.

## 4. AKI Prediction Evidence Across Oncology Settings

Table 1 summarizes the populations, outcome and baseline creatinine definitions, prediction time points and horizons, predictor availability, modeling approaches, validation designs, and reported performance of the included prediction studies. To assess clinical actionability, predictors were classified according to whether they were available before treatment or surgery, at treatment initiation or hospital admission, during treatment, or only after the principal opportunity for preventive intervention. Where available, we report calibration, AUPRC, sensitivity, specificity, positive predictive value, confidence intervals, and decision-curve or net-benefit analyses in addition to AUROC. Measures not provided by the original study are identified as “not reported.”

### 4.1. Hospitalized Cancer Patients

Oncology-specific prediction initially developed in hospitalized and routinely monitored cancer populations, where repeated laboratory testing provides dense data, but clinical trajectories remain irregular. Park et al. created a framework capable of incorporating heterogeneous EHR observations collected at variable intervals, explicitly addressing missingness and changing patient status [42]. Two studies by Li et al. used Bayesian networks to model AKI in hospitalized patients with hematologic malignancies and gastrointestinal cancers, illustrating how interpretable probabilistic methods can represent dependencies among clinical risk factors [48,49].

Scanlon et al. developed one of the largest oncology-specific models using data from more than 48,000 patients and nearly 600,000 laboratory observations. A random forest model identified patients at increased risk before clinical recognition using routinely available data. The study prospectively evaluated model performance but did not prospectively implement the model or test a prediction-triggered intervention [29]. A subsequent study externally applied this model to a general, non-cancer critical-care population using the MIMIC-IV database (*n* = 28,498), reporting an AUROC of 0.821 (vs. 0.881 in the original Christie test set) and identifying 67.3% of AKI events at a “medium or higher” risk threshold; discrimination was somewhat lower among patients with a cancer diagnosis (AUROC 0.801) than without (0.836) [62]. Nevertheless, although this evaluation was conducted in a non-oncology population and does not establish oncology-specific transportability, it remains one of the few AKI models originally derived in cancer patients to undergo external, cross-setting validation.

More recently, Guo et al. developed an explainable XGBoost model to predict AKI within 72 h of ICU admission among critically ill patients with digestive-system tumors. The model was developed using MIMIC-IV data and externally validated in three independent US and Chinese cohorts, with external AUROCs ranging from 0.616 to 0.769. Calibration was acceptable in the internal test cohort but poor across all three external cohorts, indicating a likely need for local recalibration. Decision-curve analysis showed internal net benefit across a range of thresholds, but this benefit was attenuated in the external cohorts and was largely limited to lower thresholds. The model relied primarily on general critical-illness variables, including SOFA score, sepsis, mechanical ventilation, and vasoactive-drug use, rather than cancer stage or longitudinal treatment exposures, and it has not undergone prospective clinical evaluation [50]. This study represents progress in external validation while demonstrating that transportability cannot be inferred from discrimination alone.

Collectively, hospitalized-cancer studies demonstrate the feasibility of dynamic AKI risk estimation from routine clinical data. Their next step is prospective workflow evaluation and deeper integration of cancer characteristics, treatment history, cumulative nephrotoxic exposure, and post-discharge or ambulatory data.

### 4.2. Cisplatin-Associated AKI

Cisplatin has received particularly substantial attention because nephrotoxicity is a principal dose-limiting toxicity and a defined treatment cycle creates a clear prediction window. Approximately 20–30% of patients receiving cisplatin-based chemotherapy develop AKI, depending on the population and definition used [6,27]. Common predictors include older age, pre-existing CKD, lower baseline estimated glomerular filtration rate (eGFR), higher cisplatin dose, hypoalbuminemia, hypertension, concurrent nephrotoxins, and prior kidney injury.

Motwani et al. developed and validated the first dedicated multivariable clinical score for AKI after the first cisplatin course using 4481 patients at two cancer centers. The score used four routinely available variables, including age, cisplatin dose, hypertension, and serum albumin, and achieved moderate discrimination across cancer types [43]. Subsequent work showed that the eGFR equation used at baseline can alter risk stratification, reinforcing the importance of standardized kidney-function assessment in oncology prediction models [63].

Gupta et al. later derived and externally validated a parsimonious clinical score for severe AKI following the first intravenous cisplatin administration across geographically diverse academic cancer centers. By targeting a twofold creatinine increase or kidney replacement therapy, the model focused on a less frequent but more clinically consequential outcome and represents the strongest multicenter validation among current cisplatin scores [44].

ML studies have expanded the range of variables and functional relationships considered. Okawa et al. combined artificial neural-network and gradient-boosted decision-tree methods using routinely available information from patients receiving their first cisplatin course. The model was developed in 1014 patients and evaluated in a temporally separated same-center cohort of 226 patients. Its strongest performance was observed among patients aged 65 years or older, with an AUROC of 0.78; serum albumin, body-surface area, and maximum daily cisplatin dose were among the most influential predictors. The outcome was based on Common Terminology Criteria for Adverse Events-based serum creatinine outcomes rather than KDIGO AKI, and the model lacked independent external validation [51].

Ambe et al. subsequently developed an interpretable CatBoost model using 29 clinical and laboratory measurements and cisplatin exposure variables in 1253 patients receiving their first cisplatin course, of whom 119 developed AKI. Cisplatin-associated AKI was defined using the serum creatinine component of the KDIGO criteria within 14 days after the final cisplatin administration in the first treatment course. The model achieved an AUROC of 0.78, and SHAP analysis was used to characterize the contribution of clinical and treatment-related variables to individual predictions. Nevertheless, the model did not incorporate urine-output criteria and lacked independent external validation [52].

These studies address complementary rather than directly competing objectives. Motwani and Gupta prioritize parsimonious clinical scores and stronger multicenter validation, whereas Okawa and Ambe illustrate the ability of ML to incorporate broader treatment and medication data and model nonlinear relationships. Their AUROCs should not be compared directly, however, because definitions, outcome severity, prediction windows, populations, and validation designs differ. Current evidence does not establish that ML outperforms well-developed conventional scores. Most models also remain static around the first cisplatin exposure and do not account for repeated cycles, cumulative dose, changing kidney function, or evolving nephrotoxic burden. Prospective studies are needed to determine whether risk-guided care reduces AKI while maintaining cancer-treatment intensity.

### 4.3. Contrast-Associated AKI

Contrast-enhanced computed tomography (CT) is central to cancer diagnosis, staging, response assessment, and surveillance. Patients may undergo serial contrast exposures while receiving nephrotoxic therapies or experiencing volume depletion. The term contrast-associated AKI (CA-AKI) is important because AKI after imaging is not necessarily caused by contrast, particularly when competing causes are common.

Risk tools have historically been derived in non-oncology populations, most notably the Mehran score for percutaneous coronary intervention which incorporates variables including baseline kidney function, diabetes, heart failure, and contrast volume [64]. Their applicability to CT in oncology is uncertain because cancer populations differ in treatment exposure, nutritional status, supportive medications, and imaging frequency.

Gupta et al. developed the most comprehensive oncology-specific score using 46,593 contrast-enhanced CT examinations across 25,184 patients at three academic centers. Because patients could contribute multiple CT examinations, generalized estimating equations accounted for within-patient correlation. A point score based on cancer type, kidney function, albumin, proteinuria, comorbidities, medications, and contrast volume achieved a C-statistic of 0.74 in both the development and validation samples and observed CA-AKI risk ranged from 2.2% in the lowest risk group to 32.7% in the highest-risk group [53].

This setting has a practical implementation advantage because risk can be estimated before imaging and linked to medication review, volume assessment, contrast-volume minimization, and post-imaging monitoring. However, the model was retrospectively developed and internally validated within the same health system. Although repeated examinations were handled statistically, the score remained encounter-based and did not model the number, timing, or cumulative dose of prior contrast exposures. Independent external validation and prospective evaluation of score-guided preventive pathways are therefore needed.

### 4.4. Hematopoietic Stem Cell Transplantation

HSCT is among the highest-risk oncology settings for AKI, with contemporary incidences approaching or exceeding 50% [14]. Earlier studies reported AKI in approximately 40–70% of allogeneic HSCT recipients, depending on conditioning, transplant characteristics, and the definition used [65]. Kidney injury may result from multiple overlapping mechanisms, including conditioning toxicity, sepsis, hypovolemia, sinusoidal obstruction syndrome, graft-versus-host disease, calcineurin-inhibitor toxicity, thrombotic microangiopathy, infection, and nephrotoxic antimicrobials [66].

Dedicated prediction evidence remains limited. Most studies have focused on identifying individual risk factors rather than developing and validating tools capable of supporting individualized care [65]. One notable exception is Rodrigues et al., who developed a conventional pre-transplant score incorporating pre-existing CKD, a hematopoietic cell transplantation comorbidity index of at least 2, a diagnosis of leukemia or lymphoma, and the platelet-to-lymphocyte ratio at admission [54]. The score was developed and evaluated in the same single-center retrospective cohort and has not undergone external or prospective validation. A static pre-transplantation score also cannot capture the rapidly changing post-transplantation risk.

Future models will likely require longitudinal approaches that incorporate evolving laboratory values, immunosuppressive therapy, nephrotoxic antimicrobial exposure, infectious complications, graft-versus-host disease, thrombotic microangiopathy, and other transplant-specific events. Such dynamic models could continuously update risk, facilitate earlier nephrology involvement, and support more proactive kidney-protective strategies in one of the highest-risk populations within onco-nephrology.

### 4.5. Chimeric Antigen Receptor (CAR) T-Cell Therapy-Associated AKI

CAR T-cell therapy has transformed the treatment of relapsed or refractory hematologic malignancies but is associated with a pattern of kidney injury driven largely by systemic inflammation rather than direct nephrotoxicity. Reported AKI incidence ranges from approximately 5% to 34%, and severe cytokine release syndrome (CRS), elevated baseline lactate dehydrogenase, and reduced kidney function are recurrent risk factors [67,68]. Because risk follows dynamic inflammatory changes, prediction may benefit from serial biomarkers, hemodynamics, and CRS grading. However, dedicated AI-based AKI models after CAR T-cell therapy remain unavailable, representing a clear research gap as cellular therapies expand across hematologic malignancies and solid tumors.

### 4.6. Immune Checkpoint Inhibitor-Associated AKI

ICI-associated AKI is an important but difficult-to-define complication. Reported incidence is approximately 2–5% during monotherapy and may be higher with combination regimens, but attribution varies because kidney biopsy is uncommon and competing causes are frequent [69,70]. Meraz-Muñoz et al. illustrated this distinction: all-cause AKI occurred in 16.5% of 309 patients receiving ICI, whereas only 12 cases were attributed to ICI-related nephrotoxicity by biopsy or nephrologist assessment [71].

Several studies have evaluated individualized AKI prediction. Yu et al. compared ML algorithms using random-forest recursive feature elimination [55]. Sakuragi et al. developed a LightGBM model that continuously predicted all-cause AKI within seven days and used longitudinal SHAP patterns to identify clinically interpretable clusters related to hypovolemia, drug-associated injury, and cancer cachexia [45]. More recently, Liu et al. compared nine algorithms and evaluated a SHAP-interpretable gradient-boosting model using a single-center training/test split among patients receiving PD-1/PD-L1 inhibitors [56]. Lou et al. additionally developed and externally validated a multicenter nomogram for severe AKI during anti-PD-1/PD-L1 therapy [57].

These studies demonstrate the feasibility of individualized risk estimation and explainable modeling during ICI therapy. However, models often predict all-cause AKI rather than immune-mediated nephritis, and outcome adjudication differs substantially. Future work should distinguish ICI-attributable injury from competing causes and test models prospectively across institutions and treatment combinations.

### 4.7. Targeted Therapy-Associated AKI

Despite widespread use of targeted anticancer therapies, dedicated validated AI/ML models for targeted therapy-associated AKI remain scarce. Evidence is predominantly mechanistic, pharmacovigilance-based, or observational, reflecting heterogeneous renal phenotypes across drug classes [24]. VEGF-pathway inhibitors may cause hypertension, proteinuria, and thrombotic microangiopathy; EGFR-directed therapies may cause electrolyte wasting, particularly hypomagnesemia and hypocalcemia [24]; and BRAF or MEK inhibitors have been associated with tubular injury and interstitial nephritis. Several agents, including CDK4/6, PARP, ALK, and MET inhibitors, can inhibit tubular creatinine secretion and produce pseudo-AKI without a true reduction in GFR [72].

In a real-world study of osimertinib, Lin et al. identified older age and pre-existing renal impairment as risk factors for renal injury but used conventional multivariable regression and defined injury by eGFR below 60 mL/min/1.73 m^2^ and/or proteinuria rather than KDIGO AKI [58]. The diversity of mechanisms, treatment durations, combination regimens, and pseudo-AKI makes a single pooled prediction framework unlikely to be sufficient. Future studies should use drug-specific phenotypes, longitudinal treatment data, and kidney-function measures capable of distinguishing true injury from altered creatinine handling.

### 4.8. Surgery-Associated AKI in Oncology

Major oncologic surgery is an important but procedure-specific setting for AKI prediction. Nephrectomy, cystectomy, hepatectomy, cytoreductive surgery with hyperthermic intraperitoneal chemotherapy (HIPEC), and major colorectal surgery expose patients to nephron loss, ischemia–reperfusion injury, blood loss, hemodynamic instability, dehydration, nephrotoxins, and postoperative complications [73]. Population-based data show marked heterogeneity, from less than 1% following breast cancer surgery to more than 50% after radical nephrectomy [14].

General perioperative scores were developed mainly in cardiac or nononcologic surgical populations [46,47] and do not capture systemic therapy, cancer characteristics, or nephron loss. Oncology-specific studies are emerging across several procedures. Lei et al. compared ML algorithms for AKI after liver cancer resection [59]. Lee et al. evaluated 4104 patients undergoing unilateral nephrectomy for renal cell carcinoma, where LightGBM achieved a test-set AUROC of 0.810, compared with 0.775 for an internally developed logistic-regression score and 0.626 for the SPARK index. The improvement over the internal logistic model was modest and the new models lacked external validation [60].

He et al. recently developed an interpretable XGBoost model in 1916 patients undergoing mesocolic excision across five centers. Hospitals were separated into development and independent external-validation cohorts, and the external AUROC was 0.922; SHAP highlighted tumor, operative, inflammatory, blood-loss, and transfusion-related predictors [61]. Although the He et al. model demonstrated useful discrimination, it incorporated postoperative variables that would not be available at the time of preoperative risk assessment. It may therefore support postoperative surveillance or early recognition, but its performance should not be interpreted as evidence of utility for purely preoperative prevention.

Surgical oncology offers a defined opportunity for prediction because risk can be estimated around a planned procedure. Accurate preoperative risk stratification could support individualized hydration protocols, optimization of nephrotoxic medications, early nephrology consultation, perioperative hemodynamic management, and enhanced postoperative surveillance for patients at greatest risk. Future studies should distinguish preoperative from intraoperative or postoperative prediction, validate models across health systems, and determine whether adding longitudinal cancer-treatment history improves performance and clinical utility for this population.

**Figure 3:**
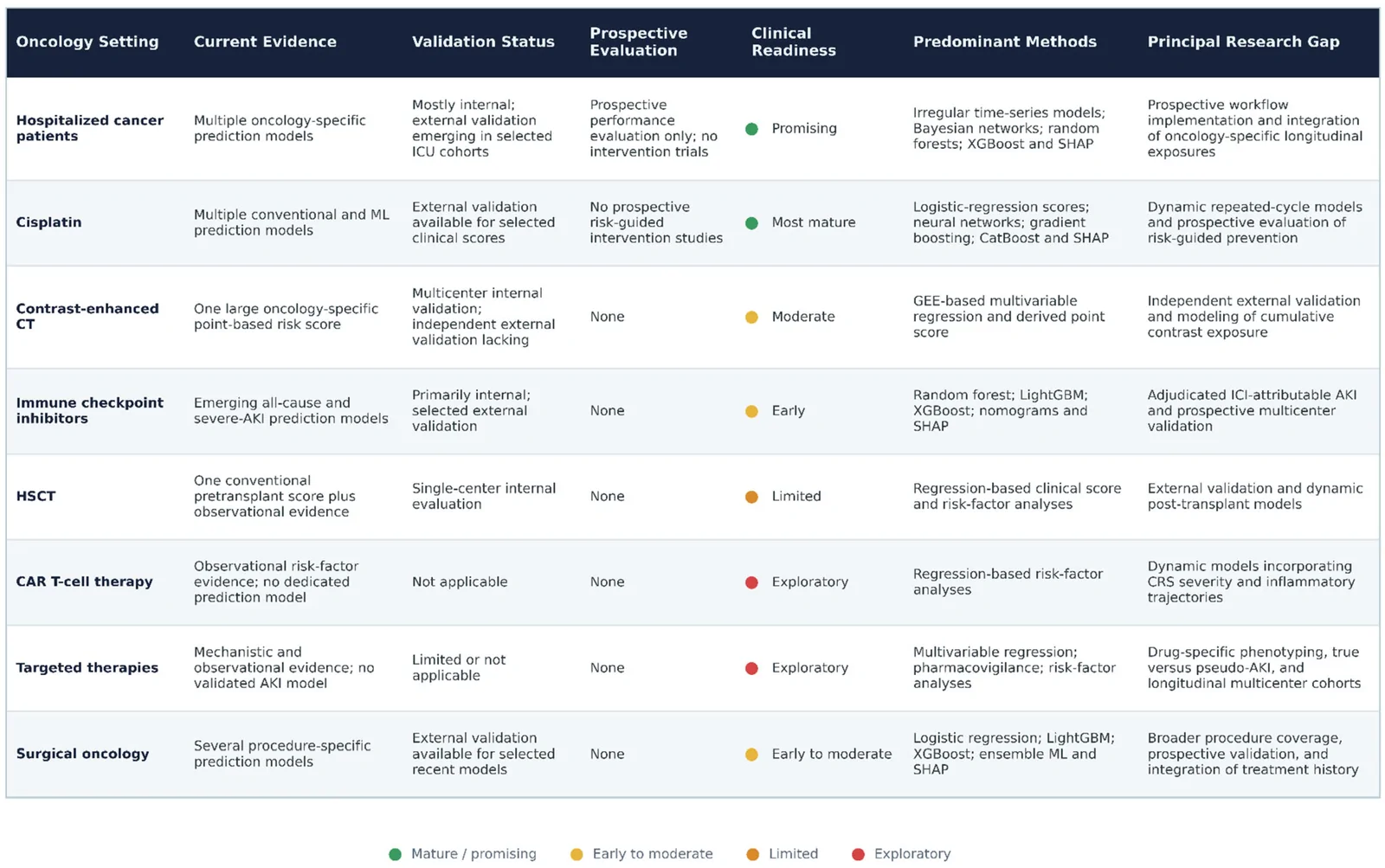
Current landscape of AKI prediction and risk-stratification evidence across major oncology settings: The figure provides a representative overview of conventional clinical risk scores, artificial intelligence and machine learning prediction models, and observational risk-factor evidence. Representative studies include models developed in hospitalized cancer populations [29,42,48,49,50,62], cisplatin-associated AKI [43,44,51,52], contrast-enhanced computed tomography [53], immune checkpoint inhibitor therapy [45,55,56,57,69,70,71], hematopoietic stem cell transplantation [54,65,66], and surgical oncology [59,60,61,73]. CAR T-cell therapy is represented by observational and risk-factor evidence [67,68], whereas targeted therapies are represented by consensus, mechanistic, and observational evidence [24,58,72]. Evidence maturity and clinical readiness reflect the extent of model development, independent validation, prospective evaluation, and clinical implementation. GEE, generalized estimating equations; HSCT, hematopoietic stem cell transplantation; ICI, immune checkpoint inhibitor; ML, machine learning; SHAP, Shapley additive explanations. Evidence-maturity categories (“mature,” “promising,” “emerging”) are descriptive author assessments, not a formal evidence-grading system.

Figure 3 synthesizes the maturity, methods, validation status, and principal gaps across these oncology settings. The figure intentionally distinguishes dedicated prediction models from observational or risk-factor evidence so that settings without validated AI/ML tools are not overstated.

## 5. Methodological Challenges and Clinical Translation

Despite promising discrimination in several settings, oncology-specific AKI prediction models have not yet been widely implemented in routine care or prospectively shown to improve kidney outcomes, cancer-treatment continuity, or survival. Most remain retrospective, single-center or health-system-specific, and few have been evaluated as part of clinical intervention. Clinical AI research more broadly shows that accurate prediction is insufficient when models are not transparently reported, externally validated, monitored, and integrated into decision-making [74,75,76,77,78].

### 5.1. Outcome Definitions and Baseline Kidney Function

Studies differ in their use of serum creatinine and urine-output criteria, staging thresholds, outcome windows, inpatient versus outpatient events, and attribution to a specific treatment. Baseline kidney function may be defined using the most recent outpatient creatinine, admission creatinine, a nadir value, or a back-calculated estimate. These choices can substantially alter AKI incidence within the same populations [79]. In supervised learning, inconsistent outcome labels introduce systematic noise and reduce comparability. Treatment-specific research must also distinguish all-cause AKI from causal phenotypes such as ICI-mediated nephritis, true contrast-associated injury, and pseudo-AKI from altered creatinine secretion.

### 5.2. Longitudinal Data, Missingness, and Treatment Context

Cancer care is distributed across hospitals, ambulatory infusion units, imaging centers, and laboratories, so EHRs may not capture complete treatment history, nephrotoxic medications, or laboratory trajectories. Monitoring is irregular and clinically driven; patterns of missingness may themselves contain information. Models that treat missingness as random may therefore introduce bias. Recurrent architectures can encode measurement intervals and missingness patterns [80], while attention-based methods can represent irregularly sampled time series [81]. Distributed multicenter approaches may also support model development without centralizing patient-level data [82]. These methods remain underused in oncology-specific AKI prediction.

A further challenge is the substantial real-world heterogeneity in pre-treatment health, cancer characteristics, treatment regimens, and care settings, which limits direct comparison across prediction studies. In addition to using narrower inclusion criteria or prespecified clinical strata, computational phenotyping may help identify more homogeneous patient groups. For example, community-detection algorithms could be applied to patient-similarity networks constructed from longitudinal clinical and treatment data to identify groups with shared risk trajectories. When combined with temporal models of evolving AKI risk, this approach could identify subgroups defined by factors such as baseline kidney reserve, treatment regimen, cumulative nephrotoxic exposure, prior AKI, or care setting. Prediction algorithms could then be evaluated within more comparable subgroups rather than across highly heterogeneous oncology populations. This approach remains generally exploratory in oncology-specific AKI prediction and requires validation, but it may provide a framework for determining when direct model comparison, recalibration, or pooling is appropriate.

### 5.3. Generalizability, Calibration, Explainability, and Fairness

External validation should assess more than just AUROC, and work to guide comparison of studies. Discrimination does not indicate whether predicted probabilities are accurate, whether performance is acceptable at clinically relevant thresholds, or whether use of the model would improve decision-making. In this way, Table 1 summarizes calibration measures, AUPRC, sensitivity, specificity, positive predictive value, confidence intervals, and decision-curve or net-benefit results wherever these were reported. Calibration may deteriorate substantially when event prevalence, treatment practice, laboratory monitoring, or patient mix differs across institutions [83]. Models should therefore report discrimination, calibration, sensitivity, positive predictive value, and net benefit in the population and workflow for which they are intended [83,84]. Interpretability may improve clinical trust, particularly when models identify patient-specific contributors to risk, but post hoc explanations do not establish causality. Fairness evaluation is similarly uncommon. Performance should be examined across age, sex, race or ethnicity where legally and ethically appropriate, socioeconomic context, cancer type, treatment regimen, and baseline kidney function to prevent models from amplifying existing disparities.

### 5.4. Implementation and Clinical Response Pathways

The value of prediction depends on what happens after a high-risk result. Alerts that are poorly timed, nonspecific, or disconnected from clinical responsibility may increase workload without improving care. The March 2026 KDIGO AKI/AKD public-review draft emphasizes external validation and standardized response pathways rather than isolated electronic alerts [23]. In oncology, actions may include medication review, hydration or hemodynamic optimization, dose assessment, contrast minimization, intensified laboratory monitoring, biomarker testing, or early nephrology consultation. The appropriate response will differ by treatment and prediction horizon.

**Figure 4:**
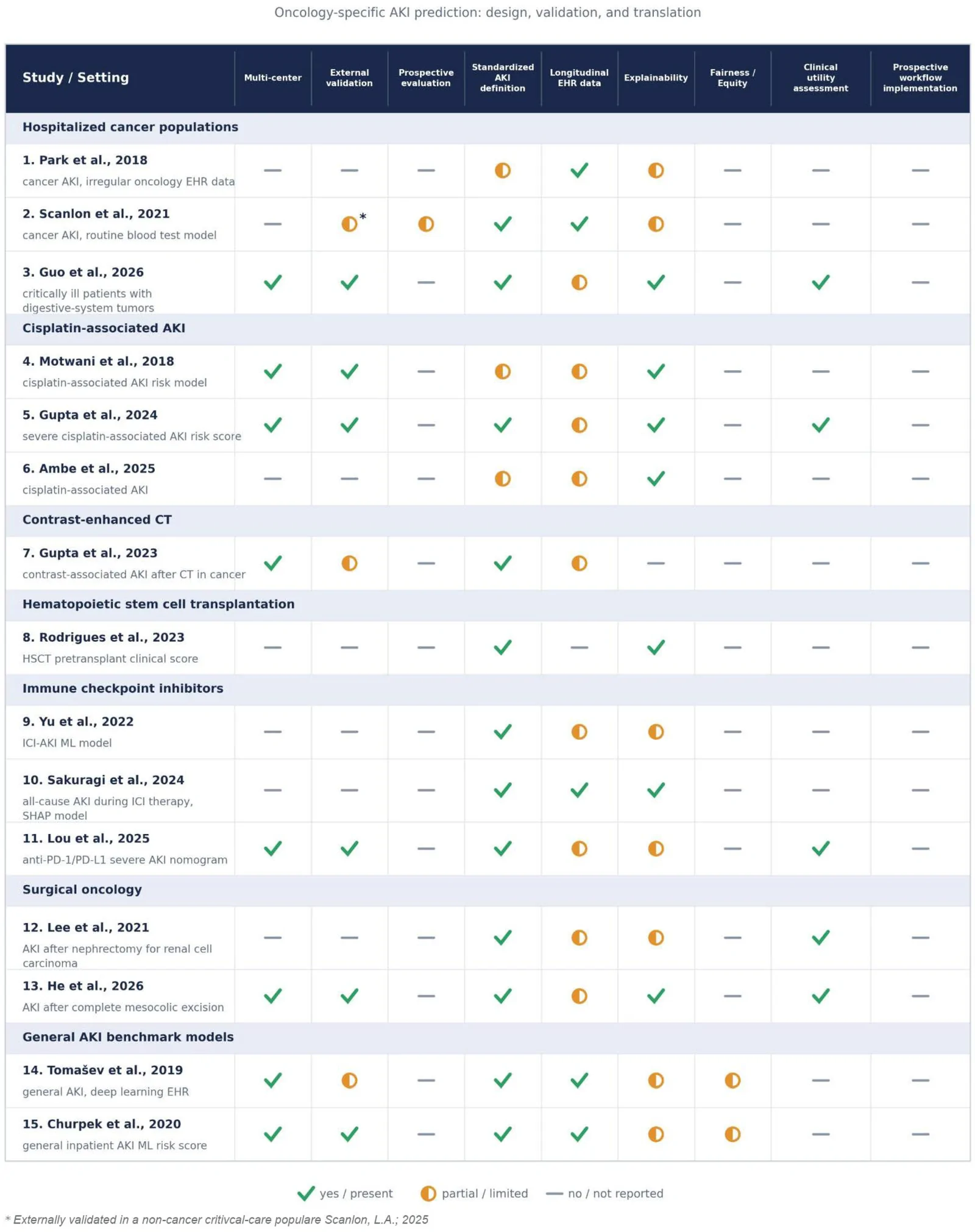
Methodological features of representative oncology-specific AKI prediction studies and selected general-AKI benchmark models: Representative studies include hospitalized cancer populations [29,42,50], cisplatin-associated AKI [43,44,52], contrast-enhanced computed tomography [53], hematopoietic stem cell transplantation [54], immune checkpoint inhibitor therapy [45,55,57], surgical oncology [60,61], and selected general-AKI benchmark models [21,22]. Check marks indicate that a feature was present; half circles indicate partial or limited reporting or evaluation; dashes indicate that the feature was absent or not reported. Clinical-utility assessment indicates whether a study evaluated the consequences of model-guided decision making through decision-curve analysis, net-benefit analysis, clinically relevant thresholds, or another formal utility assessment. The availability of a score or calculator alone was not considered evidence of clinical utility. Prospective workflow implementation indicates real-world deployment in which predictions were delivered within a clinical workflow and linked to a predefined clinical response or intervention. Prospective data collection or prospective performance evaluation without prediction-triggered clinical action was not classified as workflow implementation. None of the included studies tested whether a prediction-triggered intervention improved patient outcome. AKI, acute kidney injury; CT, computed tomography; EHR, electronic health record; HSCT, hematopoietic stem cell transplantation; ICI, immune checkpoint inhibitor; ML, machine learning; PD-1, programmed cell death protein 1; PD-L1, programmed death-ligand 1; SHAP, Shapley additive explanations.

Prospective implementation studies should therefore evaluate a complete sociotechnical intervention including data availability, model performance, alert timing, clinical acceptance, response fidelity, unintended consequences, and patient-centered outcomes. Success should be judged by prevention or mitigation of AKI, preserved kidney function, maintained cancer-treatment intensity, reduced hospitalization, and equitable benefit, not by discrimination alone. Figure 4 compares methodological features of representative oncology-specific AKI prediction studies and selected general-AKI benchmark models and illustrates persistent gaps in prospective evaluation, fairness, and real-world workflow integration.

## 6. Future Directions: Toward Precision Onco-Nephrology

Precision onco-nephrology aims to move beyond treating established kidney injury toward continuously adapting kidney-protective care as patients undergo successive treatments and accumulate risk. AI and ML may support this transition by integrating laboratory trajectories, medication exposures, imaging, treatment history, comorbidities, and evolving clinical status into updated risk estimates. Because cancer care is inherently iterative, with repeated chemotherapy cycles, immunotherapy, surgery, imaging, and supportive treatments, AI and ML-based predictive models offer an opportunity to move beyond static baseline risk assessment toward prediction that evolves alongside the patient’s clinical care. The goal is not an isolated probability, but a system that identifies why risk is changing and connects that information to an evidence-based response.

Four priorities should guide future work. First, AKI definitions, baseline kidney function ascertainment, treatment attribution, and prediction horizons should be standardized. Second, prediction should become both phenotype- and treatment-aware. Models should distinguish, where feasible, treatment-related intrinsic kidney injury from hemodynamic, infectious, structural/postrenal, and other cancer-associated mechanisms rather than treating all oncology-associated AKI as a single outcome. Longitudinal treatment data should include cumulative dose, combination and sequential regimens, nephrotoxic supportive care, procedures, evolving cancer-related complications, and recovery after previous AKI. Models should also consider whether the clinically relevant target is any AKI, recurrent AKI, severe AKI, kidney replacement therapy, or kidney injury that alters cancer treatment. Biomarkers such as cystatin C or tubular injury markers may add value where they are measured reliably but should be evaluated for incremental benefit over routine data.

Third, multicenter validation should include calibration, clinical utility, subgroup performance, and temporal updating as oncology practice changes. Explainability and fairness should be designed into evaluation rather than added after model development. Fourth, prospective trials should test whether model-guided pathways improve outcomes compared with usual care and should measure treatment continuity, patient burden, resource use, and unintended effects alongside kidney outcomes.

**Figure 5:**
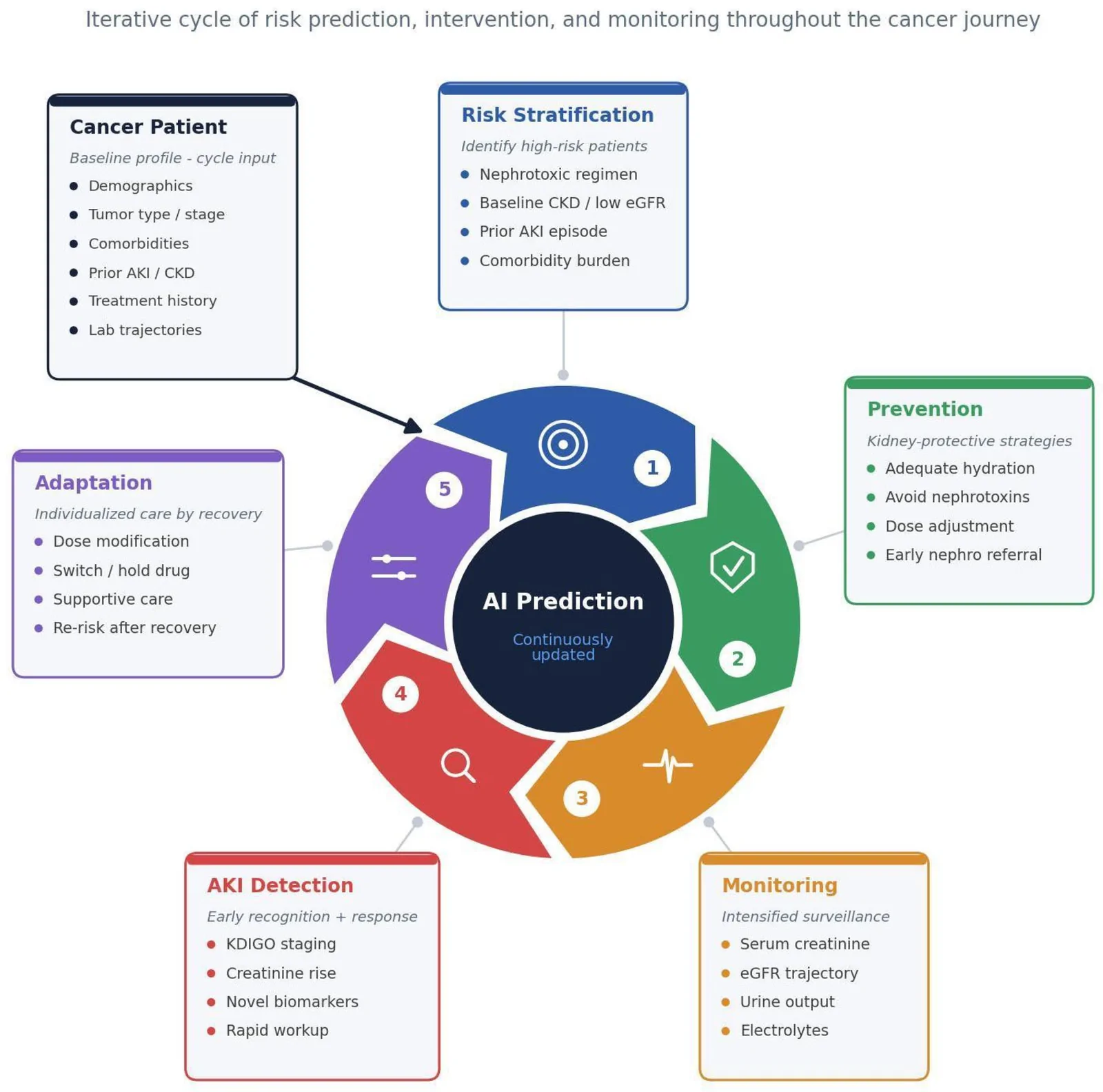
Toward precision onco-nephrology **:** Continuously updated risk prediction can support an iterative cycle of risk stratification, prevention, monitoring, AKI detection, and adaptation of cancer treatment. AKI, acute kidney injury; CKD, chronic kidney disease; eGFR, estimated glomerular filtration rate; KDIGO, Kidney Disease: Improving Global Outcomes.

Figure 5 illustrates an interactive framework in which baseline and cycle-specific data inform risk stratification, prevention, monitoring, AKI detection, and adaptation of cancer treatment. Clinical judgement remains central; prediction should augment multidisciplinary decisions by oncology, nephrology, pharmacy, radiology, surgery, nursing, and data-science teams. A learning health-system approach could then recalibrate models, monitor safety, and refine response pathways as new data accumulate.

## 7. Conclusions

AKI remains a common and clinically important complication throughout the cancer care continuum. Current evidence demonstrates the feasibility of individualized prediction in hospitalized cancer populations and in selected treatment settings, particularly cisplatin exposure, contrast-enhanced CT, ICI therapy, and procedure-specific oncologic surgery. Prediction evidence remains limited in HSCT, while evidence for CAR T-cell therapy and targeted therapies remains predominantly observational. Across settings, direct comparison is limited by heterogeneous outcomes, prediction windows, populations, and validation strategies.

Most oncology-specific models remain retrospective, and few have undergone independent external validation, rigorous calibration assessment, fairness evaluation, or prospective implementation. No model has yet shown in an interventional study that prediction-guided care improves kidney outcomes, preserves cancer-treatment continuity, or improves survival. Progress will depend on standardized definitions, high-quality longitudinal oncology data, treatment-aware and interpretable models, multicenter validation, and integration with clearly assigned clinical response pathways.

The value of AI-enabled precision onco-nephrology should ultimately be judged by outcomes that matter to patients and clinicians: preserved kidney function, safer delivery of effective cancer therapy, fewer avoidable treatment disruptions, and equitable care. The vision of precision onco-nephrology, in which kidney risk is continuously assessed and addressed throughout the cancer journey, is achievable, but will become a clinical reality only through rigorous, multidisciplinary, and clinically grounded research.

## Figures and Tables

**Table 1:** Characteristics of representative AKI prediction studies in oncology. AKI, acute kidney injury.

Study	Clinical Setting and Population	Sample Size/AKI Events	AKI Definition	Baseline Creatinine Definition	Prediction Time Point and Horizon	Predictor Availability	Candidate Predictors	Modeling Method	Validation Design	Discrimination and Classification Performance	Calibration	Clinical-Utility Assessment	Prospective Workflow Implementation
Park et al. [42]	Single center, South Korea; adult cancer inpatients and outpatients (2004–2015).	21,022 patients; number of AKI events not stated.	Modified creatinine-based KDIGO criteria; urine output not used.	Lower of the initial SCr around cancer registration or recent minimum SCr (previous 3 weeks).	At each observation; AKI within 2 weeks.	All information available before each prediction.	Demographics, clinical factors, treatments, procedures and longitudinal laboratory values.	Regression methods, random forest and MARS.	Nested cross-validation; no external validation.	Best model: precision 0.79, recall 0.75 and F1 0.76 for AKI occurrence.	NR, not reported	NR, not reported	NR, not reported
Li et al. [48]	Single center, China; hospitalized patients with hematologic malignancies (2014–2015).	2395 patients; 370 AKI events (15.4%).	KDIGO SCr criteria; urine output not used.	First SCr within 2 days of admission.	At admission; AKI during hospitalization	Admission data	Demographics, malignancy and treatment type, comorbidities, and routine laboratory values.	Group LASSO followed by a Bayesian network; logistic model used for comparison.	10-fold cross-validation; no external validation.	AUC 0.84; cross-validation AUC 0.81; logistic model AUC 0.76.	NR, not reported	NR, not reported	NR, not reported
Li et al. [49]	Single center, China; hospitalized patients with gastrointestinal cancers (2014–2015).	6495 patients; 837 AKI events in derivation cohort (14.3%).	KDIGO SCr criteria; urine output not used.	Admission SCr.	At admission; AKI during hospitalization	Admission data	Demographics, cancer and treatment type, comorbidities, and routine laboratory values.	Group LASSO and Bayesian network; compared with five other methods.	Random split: 5845 derivation and 650 validation patients; no external center.	AUC 0.82 in derivation and 0.79 in validation	NR, not reported	NR, not reported	NR, not reported
Scanlon et al. [29]	Development at a UK cancer center; later external testing in MIMIC-IV.	48,865 patients for development; 9913 prospective test patients; 28,498 in external validation.	NHS creatinine-based AKI algorithm; KDIGO outcome also tested externally.	Defined within the NHS algorithm; model also used recent and historical SCr summaries.	At each blood test; AKI within 30 days.	Current and previous blood-test results.	Routine laboratory values and derived trends; no demographics or clinical history.	Random forest with recursive feature selection and undersampling	Train/test split, prospective temporal testing, and later external validation in MIMIC-IV.	AUROC 0.88 internally and 0.82 in MIMIC-IV; sensitivity 0.43 and specificity 0.94 externally.	NR, not reported	NR, not reported	Prospective silent testing only; predictions were not used to guide care.
Guo et al. [50]	Multicenter ICU study of adults with digestive-system tumors in the USA and China.	3821 in development; external cohorts of 1670, 352 and 227 patients	KDIGO SCr or urine-output criteria; AKI within 72 h of ICU admission.	Most recent SCr in the previous 7 days; otherwise, lowest SCr in first 24 h.	First 24 h of ICU admission; AKI by 72 h	First values recorded during the first 24 h.	Seven predictors: sepsis, ventilation, vasoactive drugs, SOFA, WBC, age and potassium.	LASSO feature selection; six models compared; XGBoost selected; SHAP interpretation	70:30 split plus external validation in three cohorts.	XGBoost AUC 0.74 internally; externally AUCs 0.72, 0.77 and 0.62	Acceptable internal calibration (Brier score 0.159); poor calibration in all external cohorts	Decision curves showed net benefit across thresholds of about 10–70%; external net benefit was attenuated.	Online calculator developed; no prospective clinical evaluation
Motwani et al. [43]	Two US cancer centers; adults receiving their first course of cisplatin (2000–2016).	2118 development patients (288 AKI); 2363 validation patients (274 AKI).	SCr rise of ≥0.3 mg/dL within 14 days after cisplatin	Most recent SCr within 30 days before treatment	Before first cisplatin course; AKI within 14 days	Data available before or at treatment	Demographics, cancer site, cisplatin regimen, laboratory values, medications and comorbidities.	Multivariable logistic regression converted to a point score.	Independent validation at a second cancer center.	C-statistic about 0.68 in development and 0.66 in validation.	Acceptable fit in development; observed risk increased across score groups in validation	NR, not reported	NR, not reported
Gupta et al. [44]	Six US cancer centers; adults receiving their first dose of intravenous cisplatin (2006–2022).	11,766 derivation patients (608 AKI); 12,951 validation patients (421)	Doubling of SCr or kidney replacement therapy within 14 days of cisplatin	SCr closest to treatment within the previous 30 days.	Before first cisplatin dose; severe AKI within 14 days.	Data available before or at treatment.	Age, comorbidities, cisplatin dose, and routine blood counts and chemistry values.	Multivariable logistic regression; simplified nine-variable risk score.	Derivation at one center; external validation at five centers; bootstrap internal validation.	C-statistic 0.76 in derivation and 0.72 in external validation.	Well calibrated in the external cohort.	Decision curves showed greater net benefit than earlier cisplatin models.	No prospective clinical implementation.
Okawa et al. [51]	Single center, Japan; patients receiving first-line cisplatin chemotherapy (2006–2013).	1014 development patients (184 AKI); 226 temporal validation patients (29 AKI).	CTCAE grade 1: SCr increase >1.5 times baseline or ≥0.3 mg/dL.	Pretreatment SCr; timing not further specified.	Before first cisplatin course; AKI during that course.	Baseline and treatment data.	Sex, age, body surface area, cisplatin dose, SCr, albumin, diabetes and cardiovascular history.	Ensemble of neural networks and gradient-boosted decision trees.	Temporal validation: 2006–2012 development and 2013 validation; single center.	Overall AUC 0.67; AUC 0.78 in patients aged ≥65 years.	NR, not reported	NR, not reported	NR, not reported
Ambe et al. [52]	Single center, Japan; hospitalized adults receiving their first cisplatin course (2011–2020).	1253 patients; 119 AKI events (9.5%).	KDIGO SCr criteria within 14 days after the last cisplatin dose.	Average SCr during the 7 days before first cisplatin administration.	At the end of the first cisplatin course; AKI within 14 days.	Clinical information available by the last cisplatin dose.	Laboratory values, medications, medical history, hydration and cisplatin regimen; 29 retained variables.	CatBoost with undersampling and SHAP interpretation.	Random 80:20 development/test split with an internal validation subset; no external validation.	Test AUC 0.78; sensitivity 0.88, specificity 0.57 and F1 0.31.	NR, not reported	NR, not reported	NR, not reported
Gupta et al. [53]	Three US academic centers; adults with cancer undergoing contrast-enhanced CT (2016–2020).	25,184 patients; 46,593 CT examinations; 2682 AKI events (5.8%).	SCr increase ≥0.3 mg/dL within 48 h or ≥1.5 times baseline within 14 days.	SCr closest to CT within the previous 30 days.	Before contrast-enhanced CT; AKI within 14 days.	Data available before CT, including recent medications and treatment.	Cancer type, medications, comorbidities, kidney function, albumin, platelets, proteinuria and contrast volume.	Multivariable regression accounting for repeated examinations; point-based risk score.	Random development (30,926 examinations) and validation (15,667) sets; same three centers.	C-statistic 0.74 in both development and validation.	Good fit in validation (Hosmer-Lemeshow *p* = 0.40).	NR, not reported	No prospective clinical implementation.
Rodrigues et al. [54]	Single center, Portugal; adults undergoing autologous or allogeneic HSCT (2005–2015).	422 patients; 59.1% developed AKI by 100 days.	KDIGO creatinine and urine-output criteria.	Last SCr before admission for HSCT.	At transplantation; AKI within 100 days.	Variables available at admission for HSCT.	HCT-CI, chronic kidney disease, malignancy type and platelet-to-lymphocyte ratio.	Competing-risk survival model with backward selection; four-variable point score.	Apparent performance only; no internal or external validation.	C-statistic 0.71 for the model and 0.70 for the score.	NR, not reported	NR, not reported	NR, not reported
Yu et al. [55]	Single center, China; adults with cancer receiving immune checkpoint inhibitors (2014–2019).	1616 patients; 68 ICI-associated AKI events after chart review.	KDIGO SCr criteria; urine output not used.	Most recent SCr within 3 months before first ICI dose.	Predictors collected around ICI initiation; AKI from day 21 through end of follow-up.	Baseline data plus medications from 1 month before to 3 weeks after first dose.	Demographics, cancer and ICI type, medications, comorbidities and laboratory values; 16 retained features.	Eight methods; random-forest feature elimination; SVM selected for the reduced model.	Downsampled 70:30 train/test split with 5-fold cross-validation; no external validation.	Reduced-model AUCs 0.75–0.83; best SVM AUC 0.83.	NR, not reported	NR, not reported	NR, not reported
Sakuragi et al. [45]	Single center, Japan; patients with cancer receiving immune checkpoint inhibitors (2014–2019).	616 patients; 112 patients with at least one AKI episode (18.2%).	KDIGO SCr criteria: increase ≥0.3 mg/dL or ≥1.5 times baseline.	Baseline definition not clearly reported in the main article.	Continuous predictions at each time point; AKI within 7 days.	Current EMR data and weekly summaries from the previous 4 weeks.	287 clinical variables, including laboratory values, medications and longitudinal features.	LightGBM with SHAP explanations and clustering of patient-level prediction patterns.	Patient-level 80:20 train/test split with 5-fold tuning; no external validation.	Test AUC 0.88.	NR, not reported	NR, not reported	NR, not reported
Liu et al. [56]	Single center, China; adults treated with PD-1/PD-L1 inhibitors (2018–2024).	1663 patients; 583 AKI events in the training set (43.8%).	KDIGO SCr criteria: increase ≥26.5 µmol/L or ≥1.5 times baseline.	Lowest SCr during the 3 months before each index date.	At ICI initiation; AKI within 224 days.	Most recent baseline values within 90 days before first ICI dose.	Clinical data, laboratory values and medications; 13 features in the final model.	Nine methods compared; GBM selected and interpreted with SHAP.	Random 80:20 train/test split with internal cross-validation; no external validation.	GBM AUC 0.85 in training and 0.80 in testing.	Good calibration; test Brier score 0.185.	Decision curves showed net benefit across the evaluated thresholds.	Web calculator developed; no prospective clinical evaluation.
Lou et al. [57]	Two centers, China; adults with cancer receiving PD-1/PD-L1 antibodies (2019–2023).	1738 patients; 73 severe AKI events (4.2%).	KDIGO stage 2 or 3 AKI within 12 months; creatinine criteria only.	Pretreatment SCr; timing not further specified.	At treatment initiation; severe AKI at 90, 180 and 360 days.	Baseline clinical and laboratory data plus treatment information.	Three predictors: systolic blood pressure, serum albumin and diuretic use.	LASSO and Cox regression; three-variable nomogram.	70:30 train/test split at one center; external validation at a second center.	External C-indices 0.72, 0.77 and 0.76 at 90, 180 and 360 days.	Good agreement between predicted and observed risk in all cohorts.	Decision curves supported net clinical benefit.	No prospective clinical implementation.
Lin et al. [58]	Single-center real-world cohort, China; patients with lung cancer receiving osimertinib.	1138 patients; 215 developed renal injury (18.9%).	eGFR <60 mL/min/1.73 m^2^ and/or proteinuria; not a KDIGO AKI outcome.	SCr and eGFR measured within 3 months before osimertinib.	Risk factors assessed at baseline; renal injury during follow-up.	Baseline data only.	Age, comorbidities, blood pressure, baseline SCr/eGFR and EGFR mutation type.	Multivariable logistic regression; no prediction model or score.	No model validation.	No discrimination metrics reported.	NR, not reported	NR, not reported	NR, not reported
Lei et al. [59]	Single center, China; patients undergoing resection of primary liver cancer (2008–2015).	1173 patients; 77 AKI events (6.6%).	KDIGO SCr criteria within 7 days after surgery.	Routine preoperative SCr; timing not further specified.	Before surgery; AKI within 7 days.	Preoperative and intraoperative data.	Demographics, laboratory values, tumor features and operative factors.	Decision tree, random forest, GBM and gradient-boosted decision trees.	Random 70:30 train/test split; no external validation.	Best test AUC 0.77 for gradient-boosted decision trees.	NR, not reported	NR, not reported	NR, not reported
Lee et al. [60]	Two centers, South Korea; adults undergoing partial or radical nephrectomy for renal cell carcinoma (2003–2017).	4104 patients; 1167 AKI events (28.4%).	KDIGO SCr criteria within 7 days after surgery	Preoperative SCr; timing not further specified.	Before nephrectomy; AKI within 7 days.	Preoperative and intraoperative data.	Patient, laboratory, tumor and operative factors; nephrectomy type was most influential.	SVM, random forest, XGBoost and LightGBM; compared with two logistic scores.	Random 70:30 train/test split with 10-fold tuning; no external validation.	LightGBM AUROC 0.81 (95% CI 0.78–0.84).	LightGBM was well calibrated.	Machine learning models had greater net benefit than the SPARK score.	No prospective clinical implementation.
He et al. [61]	Five centers, China; adults with colon cancer undergoing complete mesocolic excision (2010–2020).	1916 patients; 173 AKI events (9.0%).	KDIGO SCr or urine-output criteria within 7 days after surgery.	Earliest stable SCr during the 7 days before surgery.	Perioperative prediction using data through 48 h after surgery; AKI within 7 days.	Preoperative, intraoperative and first 48-h postoperative data.	Nine factors including anemia, operative features, transfusion, tumor size, NLR and CRP.	Five methods compared; XGBoost selected and interpreted with SHAP.	70:30 internal split with cross-validation; independent hospital-based external validation.	Reported AUCs of 0.92 in training, 0.88 internally and 0.92 externally.	Good calibration internally and externally.	Decision curves showed positive net benefit.	No prospective clinical implementation.

AUC/AUROC, area under the receiver operating characteristic curve; CRP, C-reactive protein; CTCAE, NCI’s Common Terminology Criteria for Adverse Events; eGFR, estimated glomerular filtration rate; HSCT, hematopoietic stem cell transplant; ICI, immune checkpoint inhibitor; MARS, multivariate adaptive regression splines; NLR, neutrophil-to-lymphocyte ratio; SCr, serum creatinine; SHAP, Shapley additive explanations. NR indicates not reported or not assessed.

## Data Availability

No new data were created or analyzed in this study. Data sharing is not applicable to this article.

## Supplementary Materials

The following supporting information can be downloaded at: https://www.mdpi.com/article/10.3390/cancers18182937/s1, Supplementary Methods S1: Literature Identification and Evidence Prioritization; Table S1: Principal PubMed Anchor Search; Table S2: Targeted PubMed Search Concepts by Oncology Setting.
