# Supplementary material for: Prediction of Acute Kidney Injury in Oncology: From Clinical Risk Scores to AI-Enabled Precision Onco-Nephrology
Source: Cancers (Basel). 2026 Sep 10;18(18):2937. doi: 10.3390/cancers18182937 (PMC13604489; doi:10.3390/cancers18182937)
Supplement: Supplementary file 1 [file cancers-18-02937-s001.zip › cancers-4497281-supplementary.pdf]

Supplementary Material

Prediction of Acute Kidney Injury in Oncology: From Clinical Risk Scores to AI-Enabled Precision Onco-nephrology

Authors: Brena F. Sena; Alexandre Homo; Camille Fahy; Anthony Van de Putte; Bertrand Roudier; Adrien Ugon; Corinne Isnard Bagnis

Supplementary Methods S1. Literature Identification and Evidence Prioritization

This state-of-the-art narrative review was informed by iterative searches of PubMed and Google Scholar conducted through 23 July 2026, supplemented by backward and forward citation searching. The searches served as anchors to identify relevant prediction studies, terminology, treatment settings, and related bodies of evidence rather than to construct an exhaustively screened systematic-review corpus. Search concepts combined acute kidney injury with cancer and terms related to prediction, clinical risk scores, artificial intelligence, and machine learning. Targeted searches addressed cisplatin, contrast-enhanced imaging, immune checkpoint inhibitors, hematopoietic stem cell transplantation, CAR T-cell therapy, targeted anticancer therapies, and oncologic surgery. Searches were restricted to English-language publications, with no restriction on publication date. We prioritized peer-reviewed studies that directly informed oncology-specific AKI prediction, including studies that developed or evaluated conventional or AI/ML-based prediction models; studies illustrating important differences in prediction timing, predictor availability, AKI definition, validation, calibration, or clinical utility; and, where dedicated models were unavailable, cancer-specific observational studies characterizing AKI incidence and clinically relevant risk factors. Conventional scores and selected general-population AKI models were considered when they provided an important clinical or methodological benchmark. Studies were prioritized through author consensus according to their direct clinical relevance, methodological contribution, population and treatment specificity, recency, and contribution beyond evidence already represented. The resulting literature set was intended to provide a representative synthesis across major oncology settings rather than an exhaustive inventory of all publications concerning AKI in cancer. Google Scholar was used for supplementary citation discovery rather than exhaustive screening. Consistent with the narrative-review design, no formal protocol, exhaustive duplicate screening, formal risk-of-bias assessment, or meta-analysis was undertaken.

Supplementary Table S1. Principal PubMed Anchor Search

```
(
  "Acute Kidney Injury"[Mesh]
  OR "acute kidney injury"[tiab]
  OR AKI[tiab]
  OR "acute renal failure"[tiab]
  OR "acute renal injury"[tiab]
)
AND
(
  "Neoplasms"[Mesh]
  OR neoplasm*[tiab]
  OR cancer*[tiab]
  OR oncolog*[tiab]
  OR malignan*[tiab]
```

OR tumor*[tiab]
OR tumour*[tiab]
OR onconephrolog*[tiab]
OR onco-nephrolog*[tiab]
)
AND
(
"Risk Assessment"[Mesh]
OR "Artificial Intelligence"[Mesh]
OR "Machine Learning"[Mesh]
OR "Deep Learning"[Mesh]
OR "Neural Networks, Computer"[Mesh]
OR "Prediction Algorithms"[Mesh]
OR predict*[tiab]
OR "prediction model*"[tiab]
OR "predictive model*"[tiab]
OR "risk model*"[tiab]
OR "risk score*"[tiab]
OR "clinical score*"[tiab]
OR nomogram*[tiab]
OR "risk stratification"[tiab]
OR "artificial intelligence"[tiab]
OR "machine learning"[tiab]
OR "deep learning"[tiab]
OR "neural network*"[tiab]
OR "random forest*"[tiab]
OR "gradient boost*"[tiab]
OR XGBoost[tiab]
OR LightGBM[tiab]
OR "support vector machine*"[tiab]
OR "ensemble learning"[tiab]
OR "explainable AI"[tiab]
OR SHAP[tiab]
)

Supplementary Table S2. Targeted PubMed Search Concepts by Oncology Setting

AKI
(
"Acute Kidney Injury"[Mesh]
OR "acute kidney injury"[tiab]
OR AKI[tiab]
OR "acute renal failure"[tiab]
OR "acute renal injury"[tiab]
)

Prediction/risk
(

predict*[tiab]
OR model*[tiab]
OR score*[tiab]
OR nomogram*[tiab]
OR "risk factor*"[tiab]
OR "risk stratification"[tiab]
OR incidence[tiab]
)

Cisplatin/platinum therapy
(
cisplatin[tiab]
OR "platinum-based"[tiab]
OR "platinum based"[tiab]
OR "platinum compound*"[tiab]
)

Immune checkpoint inhibitor
(
"Immune Checkpoint Inhibitors"[Mesh]
OR "immune checkpoint inhibitor*"[tiab]
OR ICI[tiab]
OR "PD-1"[tiab]
OR "PD-L1"[tiab]
OR "CTLA-4"[tiab]
)

Contrast-enhanced imaging
(
"Contrast Media"[Mesh]
OR "contrast media"[tiab]
OR "contrast-enhanced"[tiab]
OR "contrast associated"[tiab]
OR "contrast-associated"[tiab]
OR "contrast induced"[tiab]
OR "contrast-induced"[tiab]
)

Hematopoietic stem cell transplantation
(
"Hematopoietic Stem Cell Transplantation"[Mesh]
OR "hematopoietic stem cell transplant*"[tiab]
OR "haematopoietic stem cell transplant*"[tiab]
OR HSCT[tiab]
OR "bone marrow transplant*"[tiab]
)

CAR-T cell therapy

(
"Chimeric Antigen Receptor T-Cell Therapy"[Mesh]
OR "CAR T-cell"[tiab]
OR "CAR-T"[tiab]
OR "chimeric antigen receptor T cell*"[tiab]
)

Targeted anticancer therapy

(
"Molecular Targeted Therapy"[Mesh]
OR "targeted therap*"[tiab]
OR "tyrosine kinase inhibitor*"[tiab]
OR "VEGF inhibitor*"[tiab]
OR "CDK4/6 inhibitor*"[tiab]
OR "MET inhibitor*"[tiab]
OR "proteasome inhibitor*"[tiab]
)

Oncology surgery

(
surg*[tiab]
OR operative[tiab]
OR perioperative[tiab]
OR postoperative[tiab]
OR nephrectom*[tiab]
OR cystectom*[tiab]
OR "oncologic surgery"[tiab]
OR "cancer surgery"[tiab]
)

Each setting-specific block was combined with the AKI block and risk/prediction block using AND. For the oncologic surgery search, the cancer block from the principal search was also included. Searches were restricted to English-language publications, with no restriction on publication date.
